# Rapid and Culture Free Identification of *Francisella* in Hare Carcasses by High-Resolution Tandem Mass Spectrometry Proteotyping

**DOI:** 10.3389/fmicb.2020.00636

**Published:** 2020-05-08

**Authors:** Natalie Witt, Sandro Andreotti, Anne Busch, Kerstin Neubert, Knut Reinert, Herbert Tomaso, David Meierhofer

**Affiliations:** ^1^Mass Spectrometry Facility, Max Planck Institute for Molecular Genetics, Berlin, Germany; ^2^Bioanalytics, Institute of Biotechnology, Technische Universität Berlin, Berlin, Germany; ^3^Bioinformatics Solution Center, Department of Mathematics and Computer Science, Freie Universität Berlin, Germany; ^4^Institute of Bacterial Infections and Zoonoses, Friedrich-Loeffler-Institut, Jena, Germany; ^5^Universitätsklinikum Jena, Friedrich-Schiller-Universität, Jena, Germany; ^6^Department of Mathematics and Computer Science, Freie Universität Berlin, Berlin, Germany

**Keywords:** *Francisella tularensis*, high-resolution tandem mass spectrometry proteotyping, proteome profiling, detection of pathogens, phylogenetic analysis

## Abstract

Zoonotic pathogens that can be transmitted via food to humans have a high potential for large-scale emergencies, comprising severe effects on public health, critical infrastructures, and the economy. In this context, the development of laboratory methods to rapidly detect zoonotic bacteria in the food supply chain, including high-resolution mass spectrometry proteotyping are needed. In this work, an optimized sample preparation method for liquid chromatography-tandem mass spectrometry (LC-MS/MS)-based proteome profiling was established for *Francisella* isolates, and a cluster analysis, as well as a phylogenetic tree, was generated to shed light on evolutionary relationships. Furthermore, this method was applied to tissues of infected hare carcasses from Germany. Even though the non-informative data outnumbered by a manifold the information of the zoonotic pathogen in the resulting proteome profiles, the standardized evaluation of MS data within an established automated analysis pipeline identified *Francisella (F.) tularensis* and, thus, could be, in principle, an applicable method to monitor food supply chains.

## Introduction

Zoonotic diseases are a biological threat to humans, and some have even the potential to be misused as biological weapons. In order to assess the epidemiological situation, knowledge about the origin and the distribution of an outbreak by fast and reliable methods is essential. Cultivation of bacteria in biosafety levels 2 and 3 laboratories is the gold standard for diagnosing these pathogens and allows subsequent typing of bacteria, e.g., with PCR assays and other DNA-based techniques. Currently, matrix-assisted laser desorption ionization-time of flight (MALDI-TOF) mass spectrometry (MS) approaches are commonly used to identify *Francisella* in an automated way at low costs in a very short time (Hubalek et al., 2004; Kilmury and Twine, 2010; Seibold et al., 2010; Durighello et al., 2014; Karatuna et al., 2016; Kasap et al., 2017). However, only pure isolates can be identified, adequate reference databases are required, and some species cannot be discriminated against due to their close phylogenetic relationship. MALDI-based proteome profiling is, thus, not capable of identifying pathogens in complex biological matrices, as only the most abundant proteins can be detected.

In contrast, high-resolution electrospray ionization (ESI) LC-MS/MS has progressed tremendously in recent years (Dworzanski et al., 2004; Cheng et al., 2016). Shotgun proteomics, in combination with bioinformatics, has enabled proteomics-based microbial identifications, even to the strain level (Dworzanski et al., 2006; Karlsson et al., 2015, 2018; Hayoun et al., 2019). In parallel with the introduction of proteomics for analyzing microorganisms, this resulted in the development of an analytical methodology called proteotyping. Proteotyping is a technique, which uses high-resolution MS and proteomic analysis to comprehensively characterize, classify, and identify microorganisms. This can, for example, include taxonomic features for identification, features important for clinical responses (e.g., biomarkers of antibiotic resistance and virulence), as well as markers for biotechnological and environmental applications (e.g., biomarkers of catabolic and anabolic pathways in cell metabolism; Karlsson et al., 2015). Proteotyping, therefore, uses an amino acid sequence list of all isoforms, which are seen as mass shifts in LC-MS/MS spectra, that have arisen from non-synonymous mutations in the genes between the species. The advantage of proteotyping over whole spectrum clustering approaches is that only mass changes associated with a particular set of allelic isoforms of the same protein are considered for phylogeny derivation (Emele et al., 2019). Other methods instead also take the presence or absence of individual masses as well as peak intensity into account, which delivers less accurate results (Zautner et al., 2015).

*F. tularensis* is a highly infectious Gram-negative bacterium causing the zoonotic disease tularemia. The Centers for Disease Control (CDC) considers this bacterium as a potential biological agent of category A, which may have a major impact on an exposed human population (Rotz et al., 2002). In Germany, most humans get infected due to contact with infected hare (*Lepus europaeus*). The clinical signs and symptoms of the disease vary depending on the route of transmission and can present as ulceroglandular, oculoglandular, oropharyngeal, or pneumonic tularemia. Cultivation of the slow-growing bacterium *F. tularensis* takes several days, and optimal growth conditions require nutrient media, such as the Cystine Heart Agar. Routine laboratories usually do not offer specific diagnostic tests for this relatively rare pathogen. Serological assays can help to establish the diagnosis in human cases and have been used to screen indicator animals, such as foxes and wild boars (Sharma et al., 2013, 2014), but food samples still require a classical approach.

**FIGURE 1:**
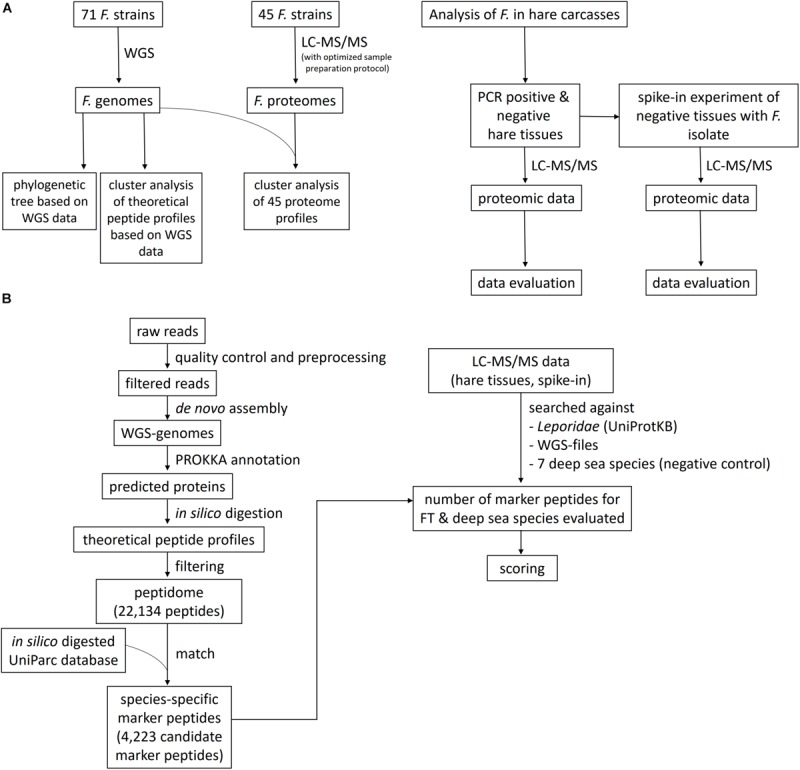
Flowchart of the study design and the bioinformatics data evaluation pipeline. **(A)** *Francisella* isolates were sequenced using whole-genome sequencing (WGS) and liquid chromatography-tandem mass spectrometry (LC-MS/MS) proteome profiling, applying an optimized sample preparation protocol for MS. A phylogenetic tree and a cluster analysis of the theoretical peptide profiles based on the WGS data were established, together with a cluster analysis of the proteome profiles. The proteome profiling method was applied to tissues of with *F. tularensis*-infected hare carcasses and to an artificial spike-in experiment, where the lower limit of detection was determined. **(B)** The bioinformatics data evaluation comprises the WGS pipeline, containing the read preprocessing, the assembly of the genomes, and the Prokka annotation. The predicted proteins were *in silico* digested, and the peptidome was matched against an *in silico* digested UniParc database. The number of these species-specific peptides was determined in the LC-MS/MS data of the hare tissues and the spike-in experiment, together with peptide hits for seven deep-sea species, which served as a negative control to determine the specificity of the applied method. A scoring function was created to evaluate the significance of the found matches.

Here, we demonstrate – as proof of principle – the use of shotgun proteome profiling by high-resolution ESI LC-MS/MS for direct evidence of *Francisella* ssp., as an exemplary representative of zoonotic bacteria, in tissues of infected hare carcasses without prior cultivation of the pathogen. Furthermore, the lower limit of detection (LLOD) of this method was determined by a spike-in of known concentrations of a *Francisella* isolate into the hare matrix. A phylogenetic tree was generated to show evolutionary relationships among *Francisella* ssp. isolates based on similarities and differences in their genetic characteristics. In addition, a cluster analysis, in which proteome data from 45 *Francisella* strains were grouped (clustered) in a way that strains in the same cluster are more similar to each other on a proteomic level than those in other groups, was applied. Therefore, an optimized LC-MS/MS sample preparation protocol was established to generate the proteome profiles of the *Francisella* isolates, along with strain-specific whole-genome sequencing (WGS) data. A scheme of the methodological strategy is outlined in Figure 1. ESI LC-MS/MS may help to analyze suspicious samples in outbreak scenarios and can be applied to inactivated specimens. This approach has the potential to screen simultaneously for a vast variety of pathogens in complex food matrices.

## Materials and Methods

### Biological Samples Derived From Carcasses of Wild Hare

*Francisella* strains (71) were selected for WGS. Institutional handling of tularemia-positive samples or cultures were done as described before (Busch et al., 2018), following the code of conduct to minimize dual-use risks (dual-use potential of life sciences research)^1^. The isolates were obtained from liver and spleen samples of hare carcasses collected by hunters in Germany between 2012 and 2018. The samples were identified and characterized at the Friedrich-Loeffler-Institut (Jena, Germany), using cultivation, MALDI-TOF MS of isolates, conventional PCR assays targeting the gene *tul4* (recognizing all three *Francisella* biogroups type A tularensis, type B holarctica, and Novicida), and WGS, as reported previously (Busch et al., 2018). The assemblies were deposited in public repositories at www.ncbi.nlm.nih.gov/bioproject (PRJNA560345, PRJNA353900, and PRJNA575140), as referenced in Supplementary Table S1.

Two primary spleen and two liver tissues derived from a total of three individual infected hare carcasses were investigated (Table 1). All four samples (16T0017, 18T0118, 18T0123, 18T0124) were autoclaved at 121°C for 30 min to allow safe handling of the material in the laboratory. In addition, spleen (*n* = 3) and liver tissue (*n* = 3) of hare carcasses that were negative for *F. tularensis* (as determined by cultivation and PCR assays) were used as negative controls (16T1200, 16T1202, 16T1188, 16T1203, 16T1205, 16T1215).

**TABLE 1:** Sample and method overview to directly identify *Francisella* in tissues collected from hare carcasses.

**Sample-nr.**	**Organ**	**Sample treatment**	**PCR**	**Culture**	**ESI-MS**
18T0118	Spleen	121°C, 30 min	Positive	Positive	Positive
16T0017	Liver	121°C, 30 min	Positive	Positive	Negative
18T0123	Liver	121°C, 30 min	Positive	Negative	Negative
18T0124	Spleen	121°C, 30 min	Positive	Negative	Negative
16T1200	Spleen	121°C, 30 min	Negative	Negative	Negative
		untreated			Negative
16T1202	Spleen	121°C, 30 min	Negative	Negative	Negative
		untreated			Negative
16T1188	Spleen	121°C, 30 min	Negative	Negative	Negative
		untreated			Negative
16T1203	Liver	121°C, 30 min	Negative	Negative	Negative
		untreated			Negative
16T1205	Liver	121°C, 30 min	Negative	Negative	Negative
		untreated			Negative
16T1215	Liver	121°C, 30 min	negative	Negative	Negative
		Untreated			Negative

**A total of four PCR-positive Francisella samples, derived from three individual hare carcasses were used. As negative controls, three PCR and cultivation-negative spleen and liver tissues were investigated. From every negative tested specimen, one part was heat inactivated, while the other part was left untreated.**

### Whole-Genome Sequencing

WGS was performed as described previously (Busch et al., 2018). In brief, the DNA was prepared from a 10 mL culture in brain heart infusion broth (Brain, Heart Infusion Broth, Sifin, Berlin, Germany). Bacterial cells were harvested after 72 h by centrifugation, and the DNA was purified using QIAGEN Genomic-tip 20/G and a QIAGEN Genomic DNA buffer set kit (Qiagen, Hilden, Germany). The DNA quality was examined using a Qubit 2.0 fluorometer (Life Technologies, Germany) and agarose gel electrophoresis. All strains were subjected only to Illumina HiSeq and/or MiSeq sequencing using the Nextera XT DNA protocol for library preparation (in house; GATC, Konstanz, and BfR, Berlin, Germany). The raw reads for data deposited in PRJNA575140 were filtered using Flexbar (version 3.0.3) to remove adapters and perform quality trimming (BWA trimming mode with a quality offset of 25). Overlapping paired-end reads were merged using FLASH2 (version 2.2) with a minimum required overlap length between reads of 50 bp. The resulting extended fragments and non-overlapping reads were assembled using SPAdes (version 3.10.0) with the options “careful” and “cov-cutoff auto”. Data were filtered excluding data with a < 5 coverage and < 500 bp in length. Prokka (version 1.14-dev) was used to identify genomic features with the inclusion of a genus-specific BLAST database of *Francisella* and subsequent translation of CDS sequences.

### Mass Spectrometry

#### Comparison of LC-MS/MS Sample Preparation Protocols for *F. tularensis*

Two sample preparation protocols, namely, the trichloroacetic acid (TCA) extraction protocol and the iST kit (PreOmics, Martinsried, Germany) were applied to the two *F. tularensis* test strains, 08T0073 and FSC237, that were inactivated by either autoclavation (121°C, 30 min) or inactivated in 75% ethanol, performed in technical duplicates.

##### TCA Extraction Protocol

The samples were prepared according to the TCA protocol as reported previously (Deatherage Kaiser et al., 2015). In brief, 40 μg of protein of each sample was pelleted for 10 min at maximum speed (21,130 × *g*) and resuspended in 0.5 mL of 20% TCA (Sigma-Aldrich, St. Louis, MO, United States) solution in water. After incubation for 24 h at −20°C, the samples were thawed and pelleted for 10 min at 21,130 × *g* at 4°C. The pellets were washed twice in 200 μl of ice-cold (−20°C) acetone (VWR, Radnor, PA, United States), the supernatant was discarded, and the pellets were dried under the fume hood. The dried pellets were resuspended for 1 h at 60°C and 400 rpm in 100 μl of lysis buffer containing 6 M urea (GE Healthcare, Chicago, IL, United States), 14.3 mM 2-mercaptoethanol (Roth, Karlsruhe, Germany) in 50 mM Tris/HCl (pH 8.5), followed by an ultrasonic treatment until complete resuspension of the pellets. Of each protein lysate, 20 μl was digested in a total volume of 150 μl – containing 40 mM ammonium bicarbonate (Sigma-Aldrich) in water, 400 ng of trypsin (Roche, Basel, Switzerland), and 400 ng of Lys C (Thermo Fisher Scientific, Waltham, MA, United State) – for 4 h at 37°C and 300 rpm. The peptides were desalted by C18 tips (Thermo Scientific) for subsequent LC-MS/MS analysis.

##### iST Kit (PreOmics)

The iST Kit (PreOmics) was used according to the manufacturer’s protocol (pelleted cells and precipitated protein, v. 2.5). For this purpose, 8 μg of each sample was pelleted for 10 min at maximum speed (21,130 × *g*), and the digestion was performed using the provided trypsin-Lys-C mixture for 4 h.

Desalted peptides from both protocols were dried in a vacuum concentrator and dissolved in either 12 μl of LC Load (iST kit) or 5% acetonitrile (ACN, VWR) and 2% formic acid (FA; TCA protocol, Fisher Chemicals, Waltham, MA, United States), briefly vortexed and sonicated in a water bath for 2 min. After centrifugation at 16,000 × *g* for 5 min, 6 μl was injected for nano-LC-MS/MS analysis. To avoid any carry-over of peptides, one wash was always run in between all individual samples in all experiments. In order to benchmark the two sample preparation methods, the RAW MS data were processed with the MaxQuant software (v.1.5.3.30) (Cox and Mann, 2008) to search against an *F. tularensis* database obtained from UniProtKB with 19,197 entries, released in 10/2017. Mass spectra were searched by MaxQuant default settings for the iST protocol and no fixed modifications for the TCA protocol.

#### Proteome Profiling of *Francisella* Isolates

Forty-five selected *Francisella* isolates were used for proteome profiling (Supplementary Table S2) to establish a cluster analysis on the proteome level. For this high-resolution MS analysis, a randomized block design was applied (Oberg and Vitek, 2009), which uses a randomized sample cohort and technical replicates to ensure the reliability of the conclusions and to assess whether the observed differences in a measurement are likely to occur by random chance and reducing bias and variance due to known sources of experimental variation. This strategy employed technical duplicates for 42 and octuplets for three strains, a total of 108 LC-MS/MS runs (see schema in Supplementary Table S3). For every strain, 30 μg of protein was processed according to the above-mentioned iST kit, and about 1 μg of the digest was injected for nano-LC-MS/MS analysis.

#### Sample Preparation of Hare Tissues

The sample preparation workflow for complex matrices was adapted as follows: The four inactivated positive hare tissues were prepared in quintuplicates, whereas the six negative tissues were split—one half was subjected to heat-inactivation at 121°C for 30 min and the other half was left untreated, prepared in triplicates.

Ten milligrams of each tissue was homogenized under denaturing conditions with a FastPrep (twice for 60 s; 6.5 m × s^–1^; MP Biomedicals, Santa Ana, CA, United States) in a buffer containing 1% (w/v) sodium deoxycholate, 10 mM Tris(2-carboxyethyl)phosphine hydrochloride, and 40 mM 2-chloroacetamide in 100 mM Tris/HCl (pH 8.5) (Kulak et al., 2014) (all purchased from Sigma-Aldrich, St. Louis, MO, United States). Following homogenization, the samples were centrifuged for 1 min (4°C, 150 × *g*) and subsequently boiled for 10 min at 95°C and 1,000 rpm in a ThermoMixer (Eppendorf, Hamburg, Germany). The samples were transferred into new tubes and sonicated for 2 min twice (UP200St, Hielscher Ultrasonics, Teltow, Germany). For each replicate, a total protein amount of 30 μg was digested for 18 h with the iST kit (PreOmics) according to the manufacturer’s protocol with the modification that the lysis was performed using the FastPrep. In order to reduce sample complexity, a three-step fractionation was carried out. The first fraction was eluted with 100 μl of SDB-RPSx1 [100 mM ammonium formate, (AF, Santa Cruz Biotechnology, Dallas, TX, United States), 40% ACN, and 0.5% FA in water], the second fraction with 100 μl of SDB-RPSx2 (150 mM AF, 60% ACN, and 0.5% FA in water; Kulak et al., 2014), and the third fraction was eluted with the elution buffer from the iST kit for 3 min at 3,800 × *g* each. All eluates were dried in a vacuum concentrator and dissolved in 60 μl of 5% ACN, 2% FA, briefly vortexed and sonicated in a water bath for 2 min. After centrifugation for 5 min at 16,000 × *g*, 6 μl per fraction was injected for nano-LC-MS/MS analysis.

The samples for the positive hare tissues were measured using complete sample randomization, whereby the fractions belonging to one sample were measured together. The negative hare samples were measured applying a randomized block design, in which the spleen and liver samples form alternating randomized blocks.

#### Spike-In of *F. tularensis* in Biological Matrices

The capability of the LC-MS/MS method, the bioinformatics pipeline, and the LLOD of *F. tularensis* in a complex matrix were tested by spike-ins of *F. tularensis* into *F. tularensis*-negative liver (16T1215) and spleen (16T1200) tissues from autoclaved hare carcasses. Therefore, 30 μg of protein lysates from both tissues were spiked with a dilution series of the *F. tularensis* strain 08T0075. Between 100 genome equivalents (GE) to 1E^10^ GE in the highest concentration were used for the dilution series in 10-fold steps, processed according to the iST protocol. One third was finally injected for LC-MS/MS analysis in three fractions.

#### Nano-LC-MS/MS Measurement

LC-MS/MS analysis was performed by nano-flow reverse-phase liquid chromatography (Dionex Ultimate 3000, Thermo Scientific) coupled online to a Q-Exactive HF Orbitrap mass spectrometer (Thermo Scientific) via a nano-electrospray ion source, as described previously (Kurschner et al., 2017). In brief, about 1 μg of desalted peptides were injected for every sample. Peptides were first loaded on a trapping column (μ-precolumn; 300 μm ID × 5 mm; C18 PepMap100; 5 μm; 100 Å; Thermo Scientific) and washed for 5 min (2% ACN, 0.1% FA, in water), before separation on a PicoFrit analytical column (75 μm ID × 55 cm long, 15 μm Tip ID (New Objectives, Woburn, MA, United States), in-house packed with 3 μm of C18 resins (Reprosil-AQ Pur, Dr. Maisch, Ammerbuch-Entringen, Germany) at a controlled temperature of 50°C. Peptides were eluted applying a non-linear 121 min gradient with a flow rate of 0.266 μl/min as follows: Peptides were first eluted for 1 min using 12% solvent B in solvent A, before increasing the concentration over 100 min to 38% solvent B. After stepping up the concentration of solvent B for 3 min to 95%, it was reduced to the starting concentration of 3.8% B. Solvent A contained 0.1% FA and solvent B 80% ACN, 0.1% FA in water. An electrospray was generated by applying 3.5 kV. The Orbitrap was operated in a data-dependent manner (m/z range of 300 to 1,750 m/z, resolution of 60,000 at m/z 200, AGC target 1E6), followed by 12 data-dependent MS/MS scans (resolution of 30,000 with a normalized collision energy of 25 eV, AGC target 5E5). In order to avoid repeated sequencing of the same peptides, a dynamic exclusion window of 30 s was used. In addition, only peptide charge states between two to eight were sequenced.

### Data Processing and Bioinformatics Analysis

#### LC-MS/MS Analysis of *Francisella* Isolates

For the 45 *Francisella* isolates, 108 LC-MS/MS RAW files were processed using tools of the open-source library OpenMS (v. 2.4) (Röst et al., 2016) and searched individually against our *Francisella* WGS files. RAW files were converted to the mzML format, and as a first preprocessing step, a mass calibration based on identified peptides was conducted. Calibrated spectra were then searched with the MS-GF+ search engine (Kim and Pevzner, 2014) and filtered at a 1% false discovery rate. For hierarchical cluster analysis, peptides belonging to the 25% highest abundant in at least three samples were selected. For this subset, the peptide incidence vectors for each sample were generated and used to compute a hierarchical clustering using Hamming distance and average linkage.

#### Identification of Species-Specific Peptides and the Specificity of the Method to Identify *F. tularensis*

Proteins predicted for assembled genomes were *in silico* digested, and the resulting theoretical peptide profiles were analyzed and screened for species-specific peptides, as outlined in Figure 1. *In silico* digestion was performed with trypsin and without missed cleavages keeping all peptides of length 8–30 amino acids. To reduce the effect of erroneous genome assemblies or annotation failures, all peptides not occurring in at least three samples were removed. From this set of 22,134 peptides (peptidome), a subset of species-specific peptides was extracted. For this purpose, the complete UniParc database (31/07/2019) with 281.8 million proteins was *in silico* digested, and the resulting peptides were matched against the set of *F. tularensis* peptides. To ensure specificity, a conservative approach was chosen by treating the isomeric amino acids leucine and isoleucine and isobaric amino acids glutamine and lysine as equivalent during the search. Peptides not matching any other species than *F. tularensis* were retained as the final set of 4,223 species-specific peptides (Supplementary Table S4).

To assess the specificity of the method to truly identify *F. tularensis* within hare tissues, we performed similar steps to obtain species-specific peptides for seven other species (*alpha proteobacterium HIMB5*, *Geobacillus* sp. 12AMOR1, *Lutibacter profundi*, *Vallitalea guaymasensis*, *Caloranaerobacter* sp. TR13, *Deferribacter desulfuricans*, *Hydrogenovibrio crunogenus*), where it is ensured that they cannot occur in hare carcasses since their natural environment is deep-sea hydrothermal vents. The peptidome for these species was generated from all available proteins in the UniprotKB and NCBI protein databases.

#### LC-MS/MS Analysis of *Francisella* in Hare Carcasses

For the identification of *Francisella* in hare carcasses, a total of 168 RAW files were processed using the same pipeline as described above and searched against all proteins of family *Leporidae* (UniProtKB with 27,234 entries, released in 02/2019), our *Francisella* specific WGS files, and the proteins for seven additional deep-sea species, impossible to occur in hare and used to assess the specificity of the method. From the resulting high-confidence peptide identifications, the number of specific peptides for *F. tularensis* and the other seven species was evaluated for each sample. To decide whether a certain number of identified species-specific peptides for a sample is significant or due to false peptide hits, we computed a score based on a simple probabilistic model that accounts for the total number of peptide matches and also for the number of species-specific peptides relative to the size of the searched database (Supplementary Definition 1). For complete preprocessing, identification, and downstream analysis, several KNIME pipelines (Berthold et al., 2008) were created to ensure portability and reproducibility, which are available upon request. The processed MS raw and KNIME output files have been deposited to the ProteomeXchange Consortium^2^ and can be found in the PRIDE depository PXD13979.

## Results

### Phylogenetic Trees Based on WGS Data and Cluster Analysis of Proteome Profiles

**FIGURE 2:**
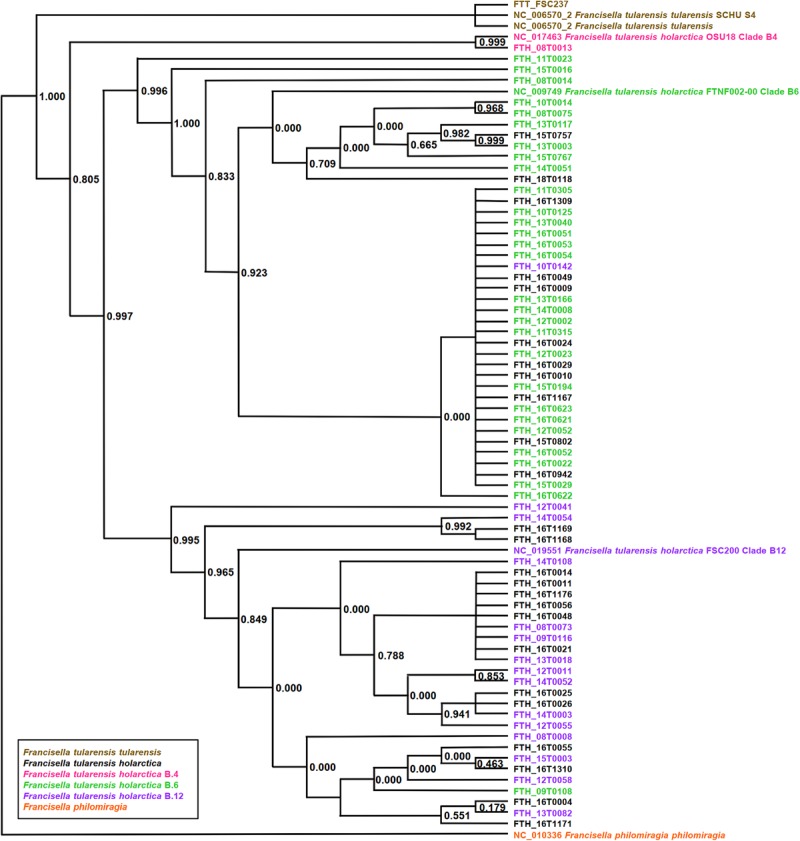
Phylogeny-based on whole-genome sequencing data from 71 *Francisella* (*F.*) isolates. SNP detection and phylogenetic analysis of genomes were carried out without genome alignment or reference genome by kSNP3.0. Assignment to clade B.4, B.6, and clade B.12 was done according to the results of real-time PCR targeting the respective loci. Colors indicate assignment to *F. tularensis holarctica* (pink to clade B.4, green to clade B.6, purple to clade B.12), *F. tularensis tularensis* (brown), and *F. philomiragia* (orange). The graphic scale equals 0.1 amino acids.

**FIGURE 3:**
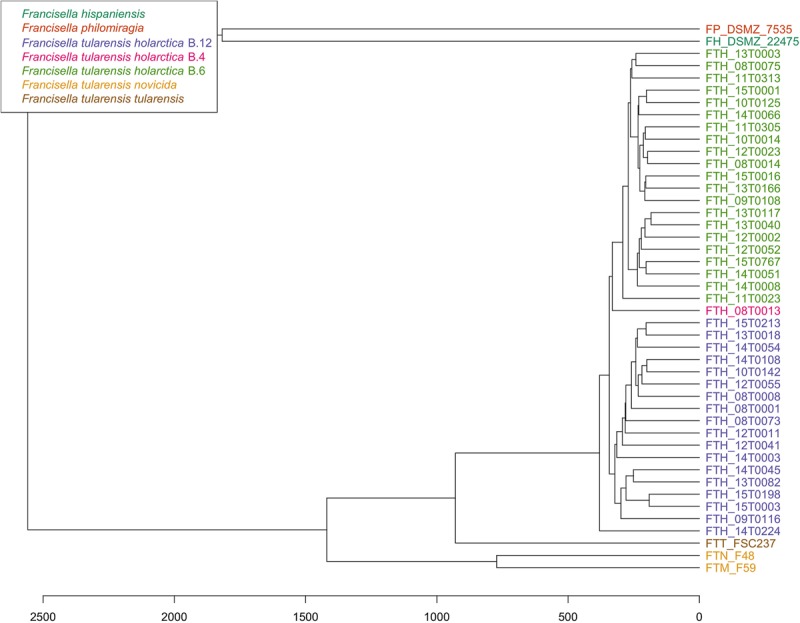
Cluster analysis of 45 *Francisella* proteome profiles. For strains with octuplets, two runs were randomly selected, and for the resulting 45 duplicates, the peptide lists were combined. Only peptides that were among the 25% highest abundant in at least three runs were considered for hierarchical clustering using Hamming distance (x-axis) and average linkage.

PhyloPhlAn, a method to assign microbial phylogeny and putative taxonomy to measure the sequence diversity of clades and subspecies (Segata et al., 2013; Seemann, 2014), was used in combination with annotation files derived from Prokka 1.14-dev, performed with standard settings to create a dendroscope (Huson et al., 2007). PhyloPhlAn includes a non-redundant database of 400 proteins generated from 3,737 genomes of all microbial taxa to assign microbial phylogeny and putative taxonomy. The software builds phylogenetic trees based on >4,600 aligned amino acid positions, mirroring more the changes in the protein sequence and, thus, functionality than on nucleotide changes that might be silent. PhyloPhlAn was able to measure the sequence diversity of all sequenced *Francisella* strains allowing even the resolution of the different clades (Figure 2). A similar clustering analysis based on all predicted tryptic peptides for the 71 isolates gave similar results (Supplementary Figure S1). It could be shown that predicted core proteome analyses lead to accurate classification of clades of *Francisella tularensis*. This raised the question if the same was true for the analysis of 45 LC-MS/MS data sets of *Francisella*. Therefore, we employed systematic and MS-based proteome profiling based on random block design, and case-specific WGS data were used to map peptides derived from the *Francisella* strains. The cluster analysis, based on the 45 proteomic profiles, assigned the isolates to the respective genetic clades as well (Figure 3) and is, thus, a versatile tool for this task.

### Method Optimization for *F. tularensis* Proteome Profiling

**FIGURE 4:**
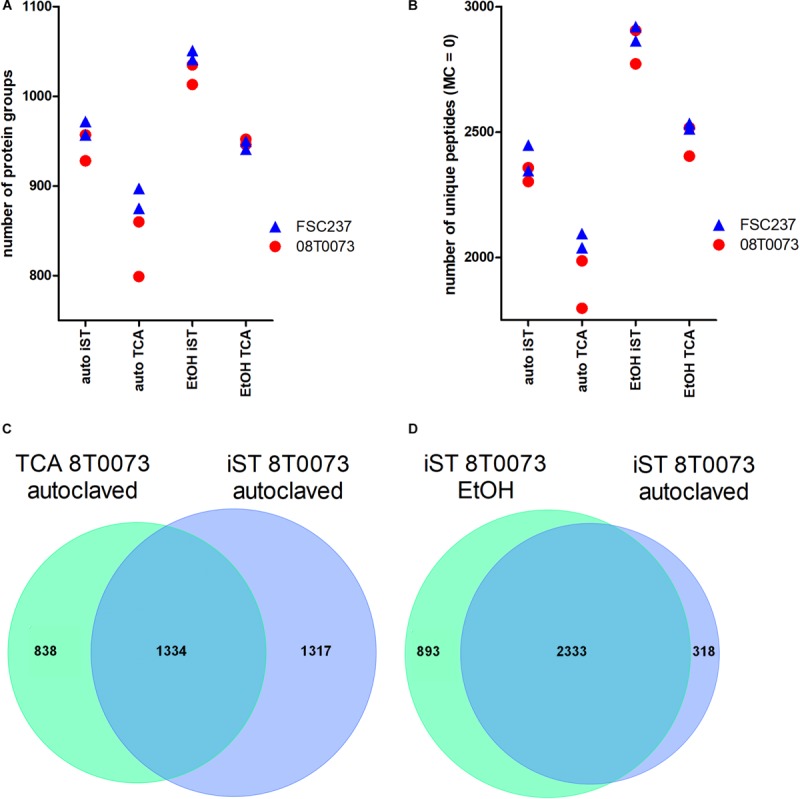
Protocol comparisons between iST and TCA sample preparation methods and autoclavation vs. ethanol inactivation to detect *F. tularensis* strains. **(A)** The identified protein groups and the unique peptides without missed cleavages (MC, **B**) were plotted for two indicated strains in technical duplicates. Furthermore, the overlap of unique peptides between both protocols for the same sample and inactivation method **(C)** and the overlap of unique peptides for the iST protocol for both inactivation methods within the same sample **(D)** are shown.

Bacterial pathogens have to be inactivated before they can be discharged from biosafety levels 2 and 3 laboratories. We asked whether the common heat inactivation at 121°C affects the subsequent LC-MS/MS analysis of proteins and, therefore, compared this method with an inactivation method in ethanol. The focus was to benchmark the number of identified unique peptides and protein groups under the premise that the number of enzymatic missed cleavages should be as low as possible. Furthermore, we aimed to identify a robust, reliable, and fast MS sample preparation protocol for *F. tularensis* that can also be easily handled in unexperienced laboratories. Ethanol inactivation of both *F. tularensis* strains resulted independent of the used sample preparation protocol in a higher average number of protein groups (Figure 4A) and unique peptides (Figure 4B) compared with heat inactivation. Even though the ethanol-inactivated samples resulted in higher proteome identification rates, the heat-inactivated samples are also applicable for MS sample preparation. This is specifically of relevance when working with highly infectious pathogens, such as *F. tularensis*, where safe handling has a high priority. Hence, inactivation by autoclavation was chosen as a default method for all further work in our study.

The comparison of the iST kit with the TCA sample preparation protocol revealed considerably better results for the iST kit in terms of the number of identified protein groups and unique peptides without tryptic missed cleavages, independent of the inactivation method (Figures 4A,B). Furthermore, the overlap of unique peptides for the TCA and the iST protocol was evaluated for the different samples and inactivation methods. The results showed that within the same inactivation method, ∼50% of all unique peptides were shared between the iST and the TCA protocol (Figure 4C). Moreover, the overlap of the unique peptides for different inactivation methods within the same digestion protocol was compared and showed that over 80% of the unique peptides were shared (Figure 4D). Thus, it is of importance to apply the same methodology for a given set of species-specific peptides. Next, we compared the ease and time of handling and the robustness of these two methods. Albeit both protocols resulted in a robust outcome, the iST kit can be performed within one working day without the need of experienced skills, whereas the TCA protocol takes twice as long and requires higher experimental acquirements. For these reasons, the iST kit was applied for all subsequent sample preparations.

### Direct Measurement of *F. tularensis* Peptides in Biological Matrices

To answer the open question, whether a direct identification of the pathogen *F. tularensis* in a complex biological matrix, such as hare tissue, is possible, we employed systematic and MS-based proteome profiling in two infected and three non-infected spleen and liver tissue samples of the hare. In one sample (18T0118) that was positive for *F. tularensis*, verified by cultivation and further characterization of the isolate (Table 1), 52 specific peptides were found in total, ranging between 32 and 36 peptides for individual replicates, and the diagnosis could be established (Supplementary Tables S5, S6A,B). In two samples that were only PCR positive (18T0123 and 18T0124), the number of bacteria in the tissue was seemingly too low, as even cultivation was not possible (Table 1). However, for sample 18T0124, five specific peptides were matched in one replicate producing a signal above noise level but did not reach significance (score < 50) and was also not consistent across the replicates. Hence, without further investigation, no unequivocal diagnosis could be established for this case.

As expected for the seven deep-sea species, only a few species-specific peptides could be matched. Although the absolute number of matched specific peptides for *V. guaymasensis* was up to 15 hits for some samples, this did not produce a significant score as the number of specific peptides for this species is more than 14 times higher than for *F. tularensis* (Supplementary Table S7). The same holds for the negative samples where neither *F. tularensis* nor any of the other species had a significant number of species-specific peptide hits (Supplementary Tables S8A,B), demonstrating the specificity of the method.

Furthermore, to resolve the influence of the inactivation procedure on the proteome data quality, e.g., protein degradation and modification at high temperatures, one part of each negative tested specimen was investigated after inactivation at 121°C for 30 min, while the other part was untreated.

This analysis was solely based on *Leporidae* and *Francisella* specific databases. The results showed that the number of identified unique peptides was higher for the untreated [26,674 ± 2,856 standard deviation (SD)], compared to the heat-inactivated (20,578 ± 3,980 SD) tissues (Supplementary Table S9). As a result, protein degradation and modifications at high temperatures do not appear to play a major role in the integrity of peptides in LC-MS/MS analysis.

### Spike-In of *F. tularensis* Into Biological Matrices

**FIGURE 5:**
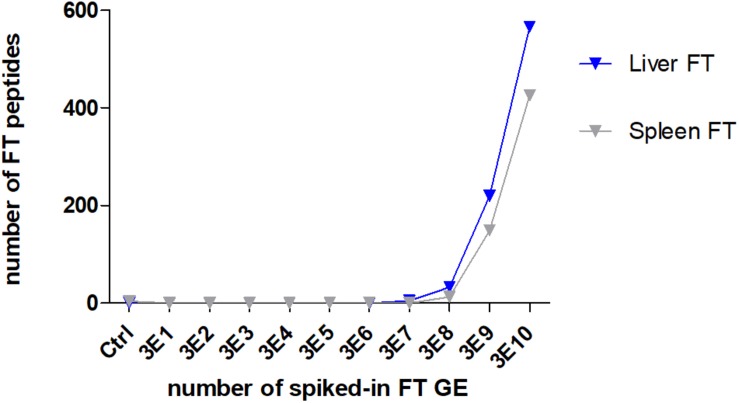
Lower limit of detection (LLOD) for an *F. tularensis* strain spiked into lysates of a liver and a spleen sample. The numbers of identified specific *F. tularensis* peptides vs. the numbers of spiked-in GE per measured sample are displayed.

To evaluate the power of the ESI proteome profiling approach for detecting *F. tularensis* in complex matrices, an artificial *F. tularensis* spike-in was performed. A similar number of identified peptides mostly derived by the matrix were identified in all samples for liver (mean 9,501 ± 872 SD) and spleen (10,531 ± 535 SD), whereas the number of *F. tularensis*-specific peptides correlated to the number of spiked-in GE (Figure 5). The LLOD of *F.* tularensis-specific peptides was for both tissues 3E8 GE, since the computed scores for the identified species-specific peptides in these samples (Supplementary Table S10) were above the significance level (>50). The scores were not significant for the other seven deep-sea species.

## Discussion

Tularemia is a rather rare disease, but nevertheless, the causative agent *F. tularensis* is of major concern because it can be used as a biological agent. Therefore, it is prudent to constantly improve the diagnostic repertoire for this pathogen. *F. tularensis* can be transmitted by contaminated aerosols, water, food, as well as via contact with infected animals and skin lesions. Contaminated hares are the most important source of human cases of tularemia in Germany, but also other food or water can be contaminated and lead to clinical presentation such as oropharyngeal tularemia. Most human cases are diagnosed using serological assays, but seroconversion takes time, which results in a diagnostic window of ∼2 weeks (Chaignat et al., 2014). In outbreak scenarios, it is important to screen potential sources as soon as the index case has been diagnosed. Fast assessment of the contaminated food items will help to identify exposed people and to offer adequate prophylactic or therapeutic antibiotic treatment.

The currently most common mass spectrometry approach to identify and classify bacteria was coined proteotyping and was originally based on MALDI-TOF instrumentation. Proteotyping achieves several tasks, e.g., the identification and quantification of expression patterns of proteins, which can be further used to identify active metabolic pathways or pathogenic patterns. In combination with genotyping, proteotyping offers a holistic characterization of microorganisms and is, as such, heavily dependent on bioinformatic analysis pipelines. The fast-growing genome reference sequence databases, as well as streamlined bioinformatics pipelines, are a major driver for the development and use of high-resolution tandem mass spectrometry and proteotyping, which will refine the microbial phylogeny and creates a basis for proteomic identification.

A main advantage of proteotyping over classical methods (e.g., serotyping, fatty acid and lipid profiling, SDS-PAGE, etc.) is that the phenotypic characteristics of the entire taxonomic range from family, genus, species, and strain are covered, as it is the case for next-generation sequencing for genotyping. There are several distinguishable bioinformatic concepts on how to analyze proteome profiles for phylogenic purposes: The abundance and presence of marker peptides can either be used alone or in addition aligned to genome reference data for the establishment of a dendrogram. A single amino acid substitution in a peptide is already efficient enough to discriminate between two closely related strains. It should be noted that the number of species-specific peptides is always based on a current reference dataset. Changes to the reference set will consequently alter the set and number of species-specific peptides.

Direct identification of bacteria may facilitate targeted antimicrobial therapy and may help to analyze outbreaks within a short time. ESI LC-MS/MS has been shown previously to be useful for identifying clinical isolates such as *Acinetobacter baumannii* and a variety of other respiratory pathogens even in clinical samples (Wang et al., 2016, 2017).

We tested a collection of *Francisella* strains from Germany and compared the proteome results with the proteome prediction based on whole-genome sequencing data (PhyloPhlAn). As described before, a distinct differentiation of *F. tularensis* ssp. *holarctica* was possible. This could be confirmed with *Francisella* and *F. tularensis*-specific peptide profiles that could be established *in silico* and could be measured in ESI LC-MS/MS. In our study, we showed that the cluster analysis of proteome profiles can also be used as a substitute for genotyping by canonical single nucleotide polymorphisms (canSNP) and core genome multilocus sequence typing (cgMLST). Even though the *F. tularensis* subsp. *holarctica* is genetically closely related, we were able to separate the clades also on the proteome level. Interestingly, the *F. tularensis* strains 09T0108 and 10T0142 clustered on the proteome level according to their clades, while the phylogenetic tree showed the contrary. This fact requires closer inspection, but resequencing excluded a sample mix up.

The method optimization for the *F. tularensis* proteome profiling revealed that the iST kit showed robust results, together with an easy and fast handling routine, even possible in unexperienced labs. Furthermore, it could be shown that heat inactivation is a suitable method for MS sample preparation, and protein degradation at high temperatures does not appear to play a significant role on the proteome data quality. Especially while working with highly infectious pathogens like *F. tularensis*, autoclavation should – for security reasons – be the method of choice for sample inactivation. The comparison between sample preparation protocols and inactivation methods for detecting *F. tularensis* revealed further that the overlap between peptides is low. Consequently, species-specific peptides have to be established by the same workflow in order to get acceptable numbers of identifiable markers.

No similar LC-MS/MS studies could be found for *F. tularensis* in the literature, where an identification of bacteria direct out of naturally infected or spiked-in tissues were identified. LC-MS/MS approaches, which use additional immunoprecipitation steps, can detect even lower numbers of colony-forming units (cfu), such as for *Yersinia pseudotuberculosis* (2 × 10^4^/mL) (Chaignat et al., 2014), or phage amplification combined with immunoprecipitation, as shown for *E. coli* (1 cfu) (Wang et al., 2016).

In our study, fast identification of the pathogen *F. tularensis* of infected hare tissues by ESI LC-MS/MS without prior cultivation was shown to be possible, and we could demonstrate that the influence of heat inactivation did not preclude identification of the pathogen, which is very important with regard to laboratory safety. From a scientific point of view, it would be desirable to further differentiate on the subspecies level, although this is not necessary for patient treatment – the prophylactic and therapeutic measures for *F. tularensis* exposures and infections are identical.

Even though the limitation of this study is the relatively small number of primary samples that were available, we could show, as proof of principle, that it is possible to identify *F. tularensis* by direct LC-MS/MS analysis of infected hare tissue without prior cultivation. This direct ESI LC-MS/MS-based bioinformatics strategy could pave the way for future analyses to characterize microorganisms.

## Conclusion

In summary, high-resolution LC-MS/MS proteome profiling has the potential to be used as a fast and versatile tool for the identification of pathogens in complex biological matrices. This method can be used to screen suspicious samples in order to determine the origin and spread of an outbreak quickly. The preliminary identification of the pathogen using ESI LC-MS/MS will especially help to choose adequate cultivation conditions to obtain bacterial isolates that can be further characterized.

Future studies with an increased number of positive samples for different pathogens will be necessary to investigate the sensitivity and generalizability of the data analysis. This also involves more elaborate scoring functions incorporating peptide ID scores, signal intensity, and corrections for strong deviations in species-specific peptide numbers or peptide compositions that affect the peptide identification.

## Data Availability Statement

The datasets generated for this study can be found in the www.ncbi.nlm.nih.gov/bioproject: PRJNA560345, PRJNA353900, and PRJNA575140 Proteomics Data: http://proteomecentral.proteomexchange.org) via the PRIDE partner repository PXD013979.

## Ethics Statement

No ethical review process was necessary, as only hare carcasses found in nature and collected by hunters were used for this study.

## Author Contributions

HT, DM, NW, and SA contributed to the conception and design of the study. NW performed the MS experiments with exception of the spike-in experiment, which was done by DM. SA, KN, AB, and KR performed the data processing and the statistical analysis. DM wrote the first draft of the manuscript. DM, NW, SA, AB, and HT wrote the sections of the manuscript. All authors contributed to the manuscript revision, and read and approved the submitted version.

## Conflict of Interest

The authors declare that the research was conducted in the absence of any commercial or financial relationships that could be construed as a potential conflict of interest.

## Footnotes

1 https://www.rki.de/EN/Content/Institute/Dual_Use/code_of_conduct.html, 2013

2 http://proteomecentral.proteomexchange.org
