# Supplementary material for: Rapid and Culture Free Identification of Francisella in Hare Carcasses by High-Resolution Tandem Mass Spectrometry Proteotyping
Source: Front Microbiol. 2020 May 8;11:636. doi: 10.3389/fmicb.2020.00636 (PMC7225524; doi:10.3389/fmicb.2020.00636)
Supplement: Supplementary file 7 [file Data_Sheet_1.docx]

Supplementary Material

Supplementary Definition 1: Scoring function

We compute the score for a given LC-MS/MS sample and target species as follows:

Let $n$ be the number of peptide spectrum matches and $n_{p}$ the number of unique peptides after FDR filtering that were identified by searching a database with a total of $d$ unique peptides. Further, let $m$ be the number of species-specific peptides for the target species and $m’$be the number of detected species-specific peptides.

Assuming the sample does not contain the target species, all $m’$ hits must be false-positives.

As the results of the database search were filtered at 1% FDR, the expected number of false-positive unique peptide identification is approximated by $\frac{n_{p}}{100}$. Assuming a uniform distribution for the false-positive hits within the database, the probability for a false-positive peptide identification to lie in the set of size *m* is $\frac{m}{d}$ and the number of hits follows a binomial distribution with $\frac{n_{p}}{100}$ trials and success probability of $\frac{m}{d}$, i.e., $m^{'}\sim B(\frac{n_{p}}{100},\frac{m}{d})$.

Hence we can estimate the probability $\pi$ to observe at least $m’$ false-positive hits to the *m* species-specific peptides from the cumulative distribution function. The final score is then given by $\left\lfloor-10 \log\pi\right\rfloor$ and we use a significance cutoff value of 50 in this study.

It must be stated, that the assumption of a uniform distribution of false-positive hits is an oversimplification and therefore we do not refer to $\pi$ as a p-value. As the FDR is defined on peptide spectrum matches, not on unique peptides, and since especially for the highly abundant background (hare) peptides there are often multiple matches to the same peptide, which are not independent, the above model will likely underestimate the expected number of hits to the target species. Furthermore, this approach does not consider differences in the composition of peptides (e.g. length, amino acid distribution) between the sets of specific peptides for different species.


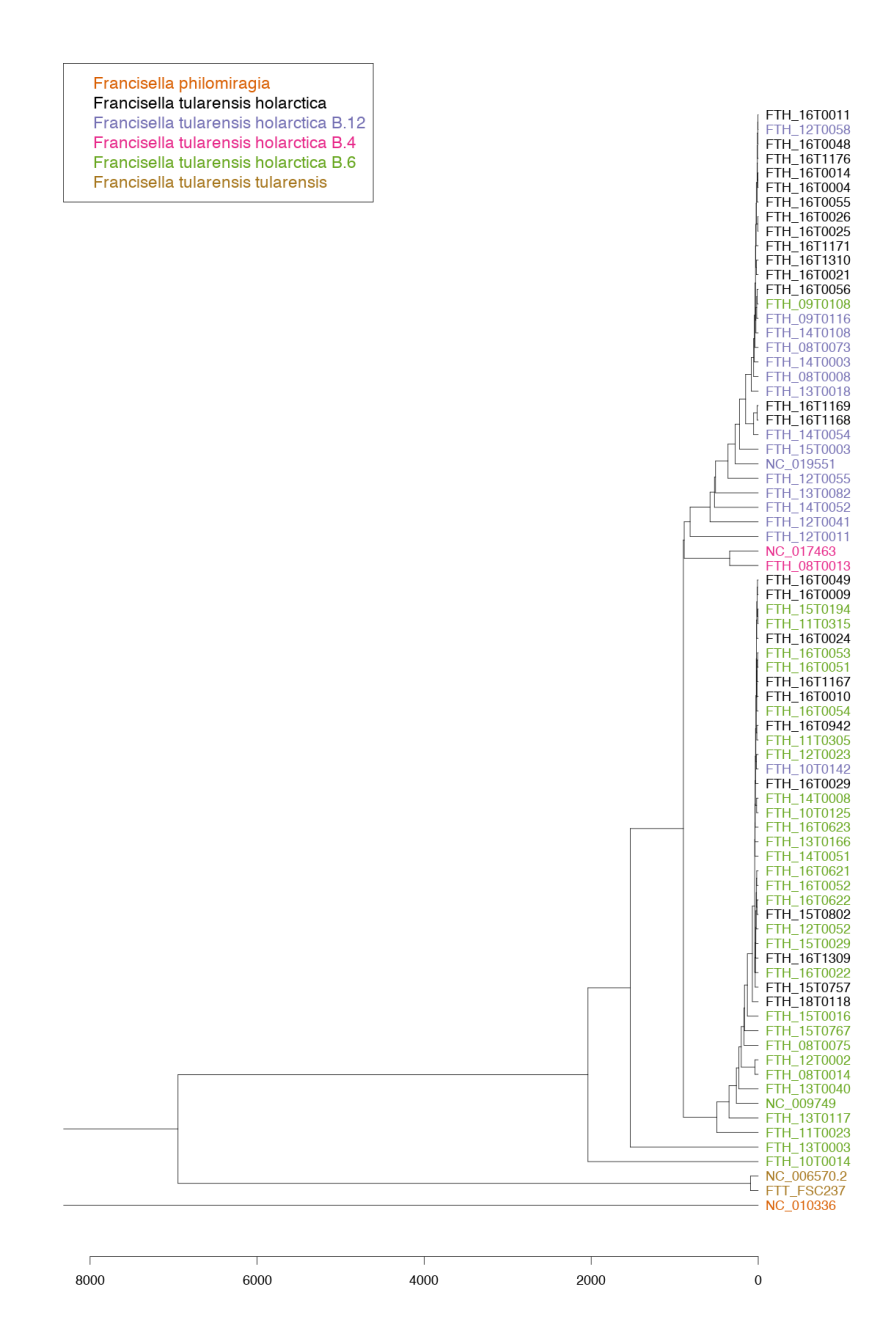


**Supplementary Figure 1: Clustering based on theoretical peptides of 71 *Francisella* strains.** *In silico* digested tryptic peptides for all 71 *F. tularensis* strains and 5 *Francisella* reference genomes (NC_006570.2, NC_009749, NC_010336, NC_017463, NC_019551) were used for hierarchical clustering. For each strain, the peptide incidence vectors (1 bit per peptide, 1 if peptide occurs for sample 0 otherwise) were generated and a hierarchical clustering using Hamming distance and average linkage was computed. For *F. tularensis holarctica* strains without clade identifier (black) currently no clade is known. The assignment of strains 09T0108 and 10T0142, contradicting their current clade annotation, is in concordance with results from PhyloPhlAn and requires closer inspection.

Supplementary Table 6A: Number of species-specific peptide hits per species and sample for positive samples. For better readability, the NCBI taxonomy IDs for the seven deep-sea species (Supplementary Table 7) were used. PSM, peptide-spectrum-matches.

| **Sample** | **1185412** | **1302151** | **1622118** | **1629723** | **197162** | **39765** | **744985** | ***F. tularensis*** | **#PSM** | **# unique peptides** |
| --- | --- | --- | --- | --- | --- | --- | --- | --- | --- | --- |
| 16T0017_1 | 8 | 3 | 0 | 1 | 6 | 3 | 0 | 1 | 43282 | 15963 |
| 16T0017_2 | 8 | 3 | 2 | 0 | 5 | 2 | 0 | 0 | 48579 | 18901 |
| 16T0017_3 | 9 | 5 | 3 | 0 | 5 | 1 | 1 | 0 | 46868 | 18234 |
| 16T0017_4 | 14 | 4 | 1 | 0 | 5 | 2 | 0 | 1 | 49287 | 18919 |
| 16T0017_5 | 13 | 2 | 2 | 0 | 2 | 2 | 0 | 1 | 44746 | 16846 |
| 18T0118_1 | 5 | 1 | 0 | 0 | 0 | 1 | 0 | 36 | 24526 | 8622 |
| 18T0118_2 | 3 | 0 | 1 | 0 | 3 | 1 | 1 | 33 | 22930 | 8490 |
| 18T0118_3 | 6 | 1 | 0 | 0 | 3 | 0 | 0 | 33 | 24106 | 8578 |
| 18T0118_4 | 6 | 1 | 0 | 0 | 3 | 0 | 0 | 32 | 24595 | 8993 |
| 18T0118_5 | 3 | 2 | 0 | 0 | 0 | 1 | 0 | 32 | 23373 | 8342 |
| 18T0123_1 | 13 | 3 | 1 | 0 | 8 | 2 | 1 | 0 | 43294 | 16748 |
| 18T0123_2 | 14 | 3 | 3 | 0 | 4 | 1 | 0 | 0 | 44600 | 17318 |
| 18T0123_3 | 13 | 3 | 3 | 0 | 4 | 3 | 1 | 0 | 43652 | 16933 |
| 18T0123_4 | 13 | 2 | 2 | 1 | 2 | 1 | 0 | 0 | 43433 | 16180 |
| 18T0123_5 | 15 | 3 | 1 | 0 | 5 | 1 | 1 | 0 | 45339 | 17308 |
| 18T0124_1 | 3 | 0 | 1 | 0 | 2 | 0 | 0 | 2 | 17028 | 6270 |
| 18T0124_2 | 4 | 0 | 0 | 0 | 1 | 0 | 1 | 2 | 12850 | 4752 |
| 18T0124_3 | 1 | 1 | 0 | 0 | 0 | 1 | 1 | 1 | 13850 | 5180 |
| 18T0124_4 | 2 | 0 | 0 | 0 | 1 | 0 | 1 | 3 | 14257 | 5574 |
| 18T0124_5 | 3 | 0 | 0 | 0 | 0 | 2 | 0 | 5 | 16517 | 6034 |

| **Sample** | **1185412** | **1302151** | **1622118** | **1629723** | **197162** | **39765** | **744985** | ***F. tularensis*** |
| --- | --- | --- | --- | --- | --- | --- | --- | --- |
| 16T0017_1 | 0 | 3 | 0 | 7 | 2 | 0 | 0 | 2 |
| 16T0017_2 | 0 | 2 | 0 | 0 | 0 | 0 | 0 | 0 |
| 16T0017_3 | 0 | 7 | 0 | 0 | 0 | 0 | 0 | 0 |
| 16T0017_4 | 1 | 4 | 0 | 0 | 0 | 0 | 0 | 1 |
| 16T0017_5 | 1 | 1 | 0 | 0 | 0 | 0 | 0 | 1 |
| 18T0118_1 | 0 | 1 | 0 | 0 | 0 | 0 | 0 | 558 |
| 18T0118_2 | 0 | 0 | 0 | 0 | 2 | 0 | 2 | 499 |
| 18T0118_3 | 1 | 1 | 0 | 0 | 1 | 0 | 0 | 497 |
| 18T0118_4 | 0 | 1 | 0 | 0 | 1 | 0 | 0 | 469 |
| 18T0118_5 | 0 | 4 | 0 | 0 | 0 | 0 | 0 | 483 |
| 18T0123_1 | 1 | 3 | 0 | 0 | 5 | 0 | 0 | 0 |
| 18T0123_2 | 1 | 2 | 0 | 0 | 0 | 0 | 0 | 0 |
| 18T0123_3 | 1 | 3 | 0 | 0 | 0 | 0 | 0 | 0 |
| 18T0123_4 | 1 | 1 | 0 | 7 | 0 | 0 | 0 | 0 |
| 18T0123_5 | 2 | 2 | 0 | 0 | 1 | 0 | 0 | 0 |
| 18T0124_1 | 0 | 0 | 0 | 0 | 1 | 0 | 0 | 12 |
| 18T0124_2 | 2 | 0 | 0 | 0 | 0 | 0 | 4 | 14 |
| 18T0124_3 | 0 | 2 | 0 | 0 | 0 | 1 | 3 | 5 |
| 18T0124_4 | 0 | 0 | 0 | 0 | 0 | 0 | 3 | 23 |
| 18T0124_5 | 0 | 0 | 0 | 0 | 0 | 3 | 0 | 45 |

Supplementary Table 6B: Scores for species-specific peptide hits per species and sample for positive samples. Scores computed according to Supplementary Definition 1. For better readability, the NCBI taxonomy IDs for the seven deep-sea species (Supplementary Table 7) were used.

| **Taxonomy ID** | **Name** | **# unique proteins** | **# unique peptides** | **# specific peptides** |
| --- | --- | --- | --- | --- |
| 744985 | *alpha proteobacterium* HIMB59 | 2803 | 30659 | 7349 |
| 1629723 | *Geobacillus sp.* 12AMOR1 | 3308 | 38252 | 963 |
| 1622118 | *Lutibacter profundi* | 2721 | 37207 | 18909 |
| 1185412 | *Vallitalea guaymasensis* | 5208 | 75909 | 61757 |
| 1302151 | *Caloranaerobacter* *sp.* TR13 | 2344 | 28716 | 11177 |
| 197162 | *Deferribacter desulfuricans* | 2539 | 34296 | 26730 |
| 39765 | *Hydrogenovibrio crunogenus* | 4791 | 44485 | 19611 |
| 263 | *Francisella tularensis* | 5170 | 22134 | 4221 |

**Supplementary Table 7: Information on analyzed species.**

Supplementary Table 8A: Number of species-specific peptide hits per species and sample for negative samples. For better readability, the NCBI taxonomy IDs for the seven deep-sea species (Supplementary Table 7) were used. PSM, peptide-spectrum-matches.

| **Sample** | **1185412** | **1302151** | **1622118** | **1629723** | **197162** | **39765** | **744985** | ***F. tularensis*** | **#PSM** | **# unique peptides** |
| --- | --- | --- | --- | --- | --- | --- | --- | --- | --- | --- |
| L_16T1203_R1_auto | 5 | 1 | 1 | 0 | 1 | 2 | 0 | 0 | 31091 | 10823 |
| L_16T1203_R1_raw | 11 | 1 | 4 | 1 | 4 | 5 | 2 | 0 | 47049 | 17211 |
| L_16T1203_R2_auto | 5 | 2 | 1 | 0 | 2 | 2 | 0 | 1 | 25272 | 8649 |
| L_16T1203_R2_raw | 10 | 2 | 3 | 1 | 5 | 4 | 0 | 0 | 44702 | 15794 |
| L_16T1203_R3_auto | 8 | 2 | 0 | 0 | 3 | 4 | 2 | 0 | 38814 | 14348 |
| L_16T1203_R3_raw | 7 | 1 | 0 | 0 | 1 | 4 | 2 | 0 | 50817 | 19420 |
| L_16T1205_R1_auto | 10 | 2 | 1 | 1 | 3 | 0 | 1 | 1 | 27289 | 9442 |
| L_16T1205_R1_raw | 8 | 3 | 3 | 0 | 5 | 3 | 0 | 0 | 47818 | 17931 |
| L_16T1205_R2_auto | 6 | 2 | 2 | 0 | 4 | 1 | 0 | 1 | 33082 | 11527 |
| L_16T1205_R2_raw | 13 | 2 | 0 | 0 | 3 | 1 | 0 | 0 | 48669 | 18212 |
| L_16T1205_R3_auto | 6 | 2 | 2 | 0 | 4 | 1 | 0 | 0 | 36177 | 13491 |
| L_16T1205_R3_raw | 9 | 2 | 3 | 0 | 7 | 3 | 0 | 0 | 47269 | 18462 |
| L_16T1215_R1_auto | 12 | 1 | 1 | 0 | 4 | 1 | 0 | 0 | 38119 | 13985 |
| L_16T1215_R1_raw | 11 | 1 | 4 | 0 | 0 | 2 | 1 | 2 | 50729 | 19713 |
| L_16T1215_R2_auto | 5 | 1 | 2 | 2 | 7 | 2 | 0 | 0 | 34233 | 12766 |
| L_16T1215_R2_raw | 7 | 3 | 3 | 0 | 4 | 4 | 3 | 0 | 49410 | 19459 |
| L_16T1215_R3_auto | 7 | 2 | 0 | 0 | 1 | 3 | 1 | 1 | 33898 | 13171 |
| L_16T1215_R3_raw | 11 | 2 | 1 | 0 | 4 | 3 | 2 | 0 | 49157 | 19484 |
| S_16T1188_R1_auto | 11 | 2 | 3 | 0 | 0 | 1 | 0 | 0 | 42109 | 18066 |
| S_16T1188_R1_raw | 15 | 2 | 1 | 0 | 4 | 2 | 2 | 0 | 49557 | 22932 |
| S_16T1188_R2_auto | 10 | 2 | 2 | 0 | 4 | 2 | 0 | 0 | 38268 | 14986 |
| S_16T1188_R2_raw | 11 | 4 | 1 | 0 | 2 | 4 | 0 | 0 | 52907 | 23648 |
| S_16T1188_R3_auto | 6 | 2 | 1 | 0 | 3 | 2 | 1 | 0 | 36942 | 14797 |
| S_16T1188_R3_raw | 12 | 2 | 1 | 0 | 3 | 2 | 2 | 0 | 47775 | 21031 |
| S_16T1200_R1_auto | 9 | 0 | 1 | 0 | 4 | 2 | 1 | 1 | 45531 | 19099 |
| S_16T1200_R1_raw | 13 | 0 | 2 | 0 | 2 | 2 | 1 | 0 | 51627 | 22945 |
| S_16T1200_R2_auto | 9 | 4 | 2 | 1 | 8 | 0 | 0 | 0 | 43114 | 18066 |
| S_16T1200_R2_raw | 13 | 2 | 1 | 0 | 1 | 1 | 1 | 0 | 44609 | 19258 |
| S_16T1200_R3_auto | 13 | 0 | 2 | 0 | 4 | 6 | 0 | 0 | 43403 | 18609 |
| S_16T1200_R3_raw | 10 | 1 | 1 | 0 | 3 | 1 | 0 | 0 | 46287 | 20907 |
| S_16T1202_R1_auto | 8 | 3 | 3 | 0 | 4 | 2 | 0 | 0 | 35586 | 15335 |
| S_16T1202_R1_raw | 9 | 1 | 5 | 0 | 4 | 1 | 0 | 1 | 40899 | 17971 |
| S_16T1202_R2_auto | 7 | 1 | 4 | 0 | 2 | 0 | 1 | 0 | 30790 | 12035 |
| S_16T1202_R2_raw | 9 | 2 | 3 | 0 | 7 | 1 | 0 | 1 | 40571 | 17153 |
| S_16T1202_R3_auto | 8 | 2 | 1 | 0 | 2 | 5 | 0 | 0 | 42605 | 18198 |
| S_16T1202_R3_raw | 7 | 3 | 2 | 0 | 1 | 0 | 2 | 0 | 36246 | 16278 |

Supplementary Table 8B: Scores for species-specific peptide hits per species and sample for negative samples. Scores computed according to Supplementary Definition 1. For better readability, the NCBI taxonomy IDs for the seven deep-sea species (Supplementary Table 7) were used.

| **Sample** | **1185412** | **1302151** | **1622118** | **1629723** | **197162** | **39765** | **744985** | ***F. tularensis*** |
| --- | --- | --- | --- | --- | --- | --- | --- | --- |
| L_16T1203_R1_auto | 0 | 0 | 0 | 0 | 0 | 0 | 0 | 0 |
| L_16T1203_R1_raw | 0 | 0 | 1 | 6 | 0 | 2 | 2 | 0 |
| L_16T1203_R2_auto | 0 | 4 | 0 | 0 | 0 | 1 | 0 | 3 |
| L_16T1203_R2_raw | 0 | 1 | 0 | 7 | 1 | 1 | 0 | 0 |
| L_16T1203_R3_auto | 0 | 1 | 0 | 0 | 0 | 2 | 3 | 0 |
| L_16T1203_R3_raw | 0 | 0 | 0 | 0 | 0 | 1 | 2 | 0 |
| L_16T1205_R1_auto | 5 | 3 | 0 | 9 | 1 | 0 | 2 | 3 |
| L_16T1205_R1_raw | 0 | 2 | 0 | 0 | 0 | 0 | 0 | 0 |
| L_16T1205_R2_auto | 0 | 2 | 0 | 0 | 1 | 0 | 0 | 3 |
| L_16T1205_R2_raw | 0 | 1 | 0 | 0 | 0 | 0 | 0 | 0 |
| L_16T1205_R3_auto | 0 | 1 | 0 | 0 | 1 | 0 | 0 | 0 |
| L_16T1205_R3_raw | 0 | 1 | 0 | 0 | 2 | 0 | 0 | 0 |
| L_16T1215_R1_auto | 2 | 0 | 0 | 0 | 1 | 0 | 0 | 0 |
| L_16T1215_R1_raw | 0 | 0 | 1 | 0 | 0 | 0 | 0 | 4 |
| L_16T1215_R2_auto | 0 | 0 | 0 | 18 | 6 | 0 | 0 | 0 |
| L_16T1215_R2_raw | 0 | 2 | 0 | 0 | 0 | 1 | 4 | 0 |
| L_16T1215_R3_auto | 0 | 2 | 0 | 0 | 0 | 1 | 1 | 2 |
| L_16T1215_R3_raw | 0 | 0 | 0 | 0 | 0 | 0 | 2 | 0 |
| S_16T1188_R1_auto | 0 | 1 | 0 | 0 | 0 | 0 | 0 | 0 |
| S_16T1188_R1_raw | 0 | 0 | 0 | 0 | 0 | 0 | 1 | 0 |
| S_16T1188_R2_auto | 0 | 1 | 0 | 0 | 0 | 0 | 0 | 0 |
| S_16T1188_R2_raw | 0 | 2 | 0 | 0 | 0 | 0 | 0 | 0 |
| S_16T1188_R3_auto | 0 | 1 | 0 | 0 | 0 | 0 | 1 | 0 |
| S_16T1188_R3_raw | 0 | 0 | 0 | 0 | 0 | 0 | 1 | 0 |
| S_16T1200_R1_auto | 0 | 0 | 0 | 0 | 0 | 0 | 0 | 1 |
| S_16T1200_R1_raw | 0 | 0 | 0 | 0 | 0 | 0 | 0 | 0 |
| S_16T1200_R2_auto | 0 | 4 | 0 | 6 | 4 | 0 | 0 | 0 |
| S_16T1200_R2_raw | 0 | 0 | 0 | 0 | 0 | 0 | 0 | 0 |
| S_16T1200_R3_auto | 0 | 0 | 0 | 0 | 0 | 3 | 0 | 0 |
| S_16T1200_R3_raw | 0 | 0 | 0 | 0 | 0 | 0 | 0 | 0 |
| S_16T1202_R1_auto | 0 | 3 | 1 | 0 | 0 | 0 | 0 | 0 |
| S_16T1202_R1_raw | 0 | 0 | 2 | 0 | 0 | 0 | 0 | 1 |
| S_16T1202_R2_auto | 0 | 0 | 3 | 0 | 0 | 0 | 1 | 0 |
| S_16T1202_R2_raw | 0 | 1 | 0 | 0 | 3 | 0 | 0 | 1 |
| S_16T1202_R3_auto | 0 | 1 | 0 | 0 | 0 | 2 | 0 | 0 |
| S_16T1202_R3_raw | 0 | 3 | 0 | 0 | 0 | 0 | 2 | 0 |

Supplementary Table 9: Comparison of autoclaved versus untreated negative hare tissues. The numbers of identified unique peptides in the negative hare tissues are shown, grouped according to sample treatment (autoclaved and untreated).

| **row ID** | **autoclaved** | **untreated** | **ratio (autoclaved/untreated)** |
| --- | --- | --- | --- |
| L_16T1203 | 16887 | 23804 | 0,7094 |
| L_16T1205 | 16713 | 25037 | 0,6675 |
| L_16T1215 | 17806 | 25185 | 0,7070 |
| S_16T1188 | 23155 | 31207 | 0,7420 |
| S_16T1200 | 26225 | 29142 | 0,8999 |
| S_16T1202 | 22680 | 25667 | 0,8836 |
| min | 16713 | 23804 | 0,6675 |
| max | 26225 | 31207 | 0,8999 |
| mean | 20578 | 26674 | 0,7682 |
| std. deviation | 3980 | 2856 | 0,0987 |
